# Brain-derived neurotrophic factor precursor in the immune system is a novel target for treating multiple sclerosis

**DOI:** 10.7150/thno.51390

**Published:** 2021-01-01

**Authors:** Zhao-Lan Hu, Cong Luo, Plinio Reinaldo Hurtado, Hui Li, Shuang Wang, Bo Hu, Jun-Mei Xu, Yang Liu, Shi-Qing Feng, Ernesto Hurtado-Perez, Kang Chen, Xin-Fu Zhou, Chang-Qi Li, Ru-Ping Dai

**Affiliations:** 1 Department of Anesthesiology, The Second Xiangya Hospital, Central South University, 139 Ren-Min Central Road, Changsha City, Hunan 410011, China.; 2 Department of Anatomy and Neurobiology, School of Basic Medical Science, Central South University, Changsha City, Hunan 410011, China.; 3 Department of Renal Medicine, Royal Adelaide Hospital, Adelaide, SA 5000, Australia.; 4 School of Pharmacy and Medical Sciences, Division of Health Sciences, University of South Australia, Adelaide, SA 5000, Australia.; 5 Department of Medical Research Center and Clinical Laboratory, Xiangya Hospital, Central South University, Changsha, Hunan 410008, China.; 6 Department of Neurology, Xiangya Hospital of Central South University, Changsha, Hunan 410008, China.; 7 Department of Orthopedics, Tianjin General Hospital, Tianjin Medical University, Tianjin 300070, China.; 8 Perinatology Research Branch, Eunice Kennedy Shriver NICHD, National Institutes of Health, Department of Obstetrics and Gynecology, Wayne State University, Detroit, Michigan 48201, USA.

**Keywords:** Multiple sclerosis, brain-derived neurotrophic factor, proBDNF, antibodies, immunotherapy, B cell, immune response

## Abstract

**Rationale:** Brain-derived neurotrophic factor precursor (proBDNF) is expressed in the central nervous system (CNS) and the immune system. However, the role of proBDNF in the pathogenesis of multiple sclerosis (MS) is unknown.

**Methods:** Peripheral blood and post-mortem brain and spinal cord specimens were obtained from multiple sclerosis patients to analyze proBDNF expression in peripheral lymphocytes and infiltrating immune cells in the lesion site. The proBDNF expression profile was also examined in the experimental autoimmune encephalomyelitis (EAE) mouse model, and polyclonal and monoclonal anti-proBDNF antibodies were used to explore their therapeutic effect in EAE. Finally, the role of proBDNF in the inflammatory immune activity of peripheral blood mononuclear cells (PBMCs) was verified *in vitro* experiments.

**Results:** High proBDNF expression was detected in the circulating lymphocytes and infiltrated inflammatory cells at the lesion sites of the brain and spinal cord in MS patients. In the EAE mouse model, proBDNF was upregulated in CNS and in circulating and splenic lymphocytes. Systemic but not intracranial administration of anti-proBDNF blocking antibodies attenuated clinical scores, limited demyelination, and inhibited proinflammatory cytokines in EAE mice. Immuno-stimulants treatment increased the proBDNF release and upregulated the expression of p75 neurotrophic receptors (p75^NTR^) in lymphocytes. The monoclonal antibody against proBDNF inhibited the inflammatory response of PBMCs upon stimulations.

**Conclusion:** The findings suggest that proBDNF from immune cells promotes the immunopathogenesis of MS. Monoclonal Ab-proB may be a promising therapeutic agent for treating MS.

## Introduction

Multiple sclerosis (MS) is a highly debilitating immune-mediated inflammatory disease characterized by neurodegeneration and autoimmune inflammation in the central nervous system (CNS) 1. MS affects more than two million people worldwide and is classified as two major phenotypes: the relapsing/remitting course and the progressive course 2. Histologically, MS is characterized by the presence of demyelinated plaques in the brain, with accompanying perivascular immune cell infiltration 1, 3. Anti-myelin-reactive CD4^+^CD8^-^ T helper type 1 (Th1) and Th17 cells are believed to play a key role in orchestrating MS-associated myelin damage 4. More recent studies have shown a critical role of B cells in MS, and B cell depletion therapy (BCDT) has shown a promising effect in MS treatment in preclinical studies and clinical trials 4, 5. Although many drugs targeting immune dysfunction have been developed in the last 20 years, their efficacies are still unsatisfactory, largely due to their side effects or limited efficacies on the primary progressive disease course 6. Therefore, continued drug discovery efforts are required to develop novel therapeutic interventions for treating MS.

MS progression is mediated by the dynamic interaction between immune-mediated tissue damage and remyelination. Among the factors known to promote remyelination are neurotrophic factors, in particular, brain-derived neurotrophic factor (BDNF) 7, which is widely expressed in the CNS and exerts neuroprotective effects, such as neuronal survival, axonal growth, and myelination 8, 9. Also, BDNF is expressed in infiltrating immune cells in MS brain lesions 10. In experimental autoimmune encephalomyelitis (EAE), a mouse model of MS, BDNF overexpression in T cells attenuates symptoms and reduces axonal damage 11. However, one study showed that BDNF derived from the CNS, but not the immune system, mediated axonal protective effects in the early phase of autoimmune demyelination 12. These studies suggested a mild neuroprotective effect of mature BDNF in the immune system during the development of MS.

BDNF is initially synthesized as a pre-pro neurotrophin and then cleaved into BDNF precursor protein (proBDNF) and can be converted into mature BDNF in the trans-Golgi by Furin or within vesicles by pro-protein convertase 1 13. ProBDNF can also be secreted and subsequently cleaved by metalloproteinases and/or plasmin, giving rise to mature BDNF extracellularly 14. In the nervous system, proBDNF binds to its high-affinity receptor, p75 pan-neurotrophin receptor (p75^NTR^), and co-receptor, sortilin, to exert opposing functions to those of mature BDNF, such as triggering neuronal apoptosis, axon pruning, neurite collapse 15, and negatively regulating neurogenesis 16 and synaptic plasticity 15. Besides its expression in the CNS, proBDNF is also expressed in immune cells such as monocytes and B cells 17-19. ProBDNF was found to play a role in regulating the functions of macrophages/microglia in neuroinflammation induced by chronic *Toxoplasma gondii* infection 20.

We have previously shown that proBDNF was released from infiltrating macrophages as a critical mediator of neuroinflammation in spinal cord injury and inflammatory pain, the activity of which could be significantly attenuated by the administration of anti-proBDNF blocking antibodies (Ab-proB) 21, 22. Increased proBDNF in monocytes/macrophages also contributed to the inflammatory response in patients with type A aortic dissection disease with intensive systemic inflammation 23. We further found that lipopolysaccharide (LPS) injection caused the upregulation of proBDNF in T cells in the immune system 24, 25. Increased proBDNF in CD4^+^ T cells was implicated in the down-regulation of meningeal CD4^+^ T cells and the pathogenesis of sepsis-associated encephalopathy 25. These findings strongly suggested the critical role of proBDNF signaling in the immune system in the pathogenesis of immune-mediated inflammatory diseases such as MS.

In the present study, we showed that proBDNF signaling was activated in circulating lymphocytes and infiltrated inflammatory cells in the spinal cord and brain of MS patients and EAE mice. Systemic but not intracranial administration of Ab-proB attenuated EAE progression by improving clinical scores, limiting demyelination, and inhibiting proinflammatory cytokine gene activation. Stimulation of human peripheral blood mononuclear cells (PBMCs) increased proBDNF release and upregulated p75^NTR^ expression in the lymphocytes. A monoclonal antibody against proBDNF inhibited the inflammatory response of PBMCs to stimulation. These results suggested that upregulated proBDNF in the immune system is a detrimental factor involved in the pathogenesis of EAE in mice, and its blockade is a promising strategy for MS treatment.

## Materials and Methods

### Human subjects

The human autopsy brain and spinal cord specimens were obtained from the Australia MS bank. The collection of human peripheral blood samples from MS patients and healthy volunteers was approved by the Ethics Committee of the Second Xiangya Hospital and registered in Chinese Clinical trial (ChiCTR1900021328). Written informed consent was obtained from all participants who provided serum in this study. The information of these patients obtained from the MS bank is presented in the supplemental files.

### Reagents

ProBDNF protein and sheep polyclonal anti-proBDNF antibody were developed and characterized previously by Prof. Zhou's laboratory 21, 26. The humanized anti-human proBDNF monoclonal antibody was developed by Shanghai Yile Biotechnology Company. The myelin oligodendrocyte glycoprotein (MOG 35-55 (MW 2581.98; amino acid sequence MEVGWYRSPFSRV VHLYRNGK) was synthesized by Meilian Biochem, Ltd., China. Other commercial antibodies used in this article are listed in Supplementary Table 3.

### EAE model

The EAE mouse model was established, as previously described 27. C57BL/6J mice were obtained from Central South University Animal Services (Changsha, China). The mice were housed 5-6 per cage with food and water *ad libitum* and kept in a 12:12 light: dark cycle, 21 ± 1℃ temperature and 50 ± 10% humidity. All experimental protocols were approved by the Animal Care and Use Committee of Central South University.

C57BL/6J mice were anesthetized by sevoflurane, and then subcutaneously injected with 100 μL MOG35-55 (3 mg/mL)/IFA (H37RA 4 mg/mL) (catalog: 231141, Difco Laboratories, USA) into the two sides of the upper part of the back on day 0. The mice were intraperitoneally (*i.p.*) injected with 50 μL of pertussis toxin (PTX) (0.5 μg/100 μL) (catalog: 180, List Biological Labs, Inc.). Forty-eight hours after the injection, the mice were injected with 50 μL of PTX for a second time. On day 7 after injection, the mice were administered with 100 μL of MOG35-55 (3 mg/mL)/IFA (H37RA 4 mg/mL) into the two sides of the lower back again. For induction of recombinant human MOG (rhMOG, kindly provided by Yile Biotechnology Company)-induced EAE, C57BL/6J mice were subcutaneously injected with 100 µg rhMOG emulsified in complete Freund's adjuvant (CFA, catalog: 344289, Sigma, USA) containing 200 µg heat-killed Mycobacterium tuberculosis (Mtb) H37RA on day 0. Additionally, mice received *i.p.* 200 ng PTX in 0.2 mL phosphate-buffered saline (PBS) on days 0 and 2. The mice *i.p.* injected with 50 μL of PBS or CFA on day 0 and day 7 were used as controls. The EAE clinical neurological deficits, including the behavior, weight changes and onset date, were monitored daily by double-blind observers for evaluating the clinical neurological function score of EAE mice. The clinical scoring criteria were set as follows: 0 point: asymptomatic; 1 point: sagging of the mouse tail; 2 points: weakness or mopping of the unilateral or bilateral hind limbs, or mild ataxia; 3 points: severe weakness of the hind limbs or severe ataxia; 4 points: quadriplegia with urinary incontinence; 5 points: pre-dying state.

### PcAb-proB or mAb-proB administration

To evaluate proBDNF effects on EAE, anti-proBDNF antibody was i.p. injected (0.08 μg/μL per g of body weight for sheep polyclonal anti-proBDNF blocking antibody (PcAb-proB); 10 μg, 30 μg, or 100 μg in 100 μL volume for monoclonal Ab-proB (mAb-proB) or IgG4) or injected into lateral ventricles (1 μg/μL, 1 μL for PcAb-proB) of EAE mice (n = 10 for each group). The injection schedule varied for early onset (on 9, 13, and 17 days after immunization) or late onset (on 17, 21, and 25 days after immunization) of EAE (Figure 3) as described previously 28, 29. The mice injected with an equal volume of sheep IgG or IgG4 were used as controls (n = 30). Lyophilized Ab-proB or isotype control from Shanghai Yile Biotechnology Company was reconstituted in sterile deionized water to a final concentration of 1 mg/mL and solubilized for 30 min at room temperature with occasional gentle mixing. For intracerebroventricular (i.c.v.) injection, mice were placed in a stereotaxic apparatus (RWD, Shenzhen, China) with bregma and lambda at horizontal level after anesthetizing with an intraperitoneal injection of pentobarbital sodium (40 mg/kg). Stereo positioning coordinates of bregma of anteroposterior -0.1 mm, mediolateral ± 1.0 mm were used for bilateral stainless cannulas implantation with a depth of 2 mm. After surgery, the mice were allowed to recover for at least 7 days before EAE induction. Ab-proB or IgG was injected into the cerebroventricular region through an injection needle that was inserted into the guide cannula at different time points as indicated.

### Histological staining

For histopathological analysis, the mice were anesthetized with sevoflurane inhalation. Spinal cords (L4-L6) were removed and fixed with 4% paraformaldehyde and embedded in paraffin. The 4 μm thick sections were stained with Luxol Fast Blue (LFB, catalog: L0294, Sigma, USA) for demyelination analysis and stained with H&E for infiltrated inflammatory cells analysis by optical microscope (Nikon ECLIPSE 80i, Nikon Corporation, Tokyo, Japan), as previously described 30.

### Immunofluorescence

The 4 μm thick spinal cord or spleen sections were permeabilized with 0.5% Triton-X-100 in PBS for 20 min and blocked with non-fat 5% BSA in tris-buffered saline for 60 min at 37 ˚C, incubated with primary antibodies (anti-proBDNF antibody, 1:200; anti-CD3, 1:200; anti-CD4, 1:200; anti-CD8, 1:200; anti-CD45R/B220, and 1:200; anti-MBP antibody) at 4 ˚C overnight. The sections were incubated with appropriate secondary antibodies at 37 ˚C for 1 h. The coverslips were stained with DAPI (1:2,000) for 2 min at room temperature and mounted on slides. Immunofluorescence images were acquired using a fluorescence microscope (Nikon ECLIPSE 80i, Nikon Corporation, Tokyo, Japan).

### Flow cytometry

To measure the proBDNF and p75^NTR^ expression in PBMCs and spleen. Fresh spleens were minced and resuspended in PBS, filtered through a 40 µm cell strainer, and centrifuged at 350 g for 10 min at 4 ˚C. Cells were washed with PBS and re-suspended in 2 mL PBS (~1-5×10^7^ cells/mL). The PBMCs of mice were collected by using Ficoll (catalog: 17-5446-02, GE Healthcare, USA). Human PBMCs were isolated from peripheral blood samples of healthy donors (HDs) and MS patients by lymphoprep separation liquid (catalog: 07851, Stemcell Technology, Norway). Freshly isolated PBMCs or splenocytes were then counted manually with a hemocytometer and cell viability was measured by trypan blue (> 96%). After blocking with Human TruStainFcX (Fc Receptor Blocking Solution) or TruStainFcX (anti-mouse CD16/32) antibody for 10 min at room temperature, cells were washed with 1 × PBS and centrifuged at 350 g for 5 min. Cells were then stained with a fluorescently-conjugated antibody cocktail at 4 °C for 30 min as follows: PE/Cyanine7 anti-human CD3, PerCP/Cy5.5 anti-human CD4, BV510 anti-human CD8, APC anti-human CD19 and PE anti-human and mouse CD271 for human PBMCs; BV421 anti-mouse CD3, APC-Cy7 anti-mouse CD4, PE/Cyanine7 anti-mouse CD8, APC anti-mouse CD19, and PE anti-human and mouse CD271 for mice PBMCs or splenocytes. Cells were fixed with fixation buffer for intracellular staining and then permeabilized with 1×permeabilization buffer (catalog: 00-8333, Invitrogen, USA) at 4 °C for 30 min. Next, the cells were incubated with the sheep-anti-human proBDNF polyclonal antibody (1:100) used by our laboratory previously 22 or sheep IgG control (1:100) at 4 °C for 30 min, followed by staining with Fluorescein isothiocyanate (FITC)-donkey anti-sheep IgG H&L. After rinsing, cells were read on the flow cytometer (Cytotek, USA) and data were analyzed with FlowJo vX0.7 software. Nonspecific binding of secondary antibodies was quantified, and a fluorescence signal was subtracted from the values of experimental groups. Single stained cells or OneComp eBeads (catalog: 01-1111-42, eBioscience, CA) were used for compensation calculations. Unstained cells, cells with 7-AAD, and fluorescence minus one (FMO) controls were used for cytometry and gating setup.

### Quantitative real-time PCR (qRT-PCR)

Total RNA was extracted from the spleen and spinal cord tissues. The cDNA was obtained using the reverse transcription kit (catalog: K1621, Thermo Fisher Scientific, USA) and qRT-PCR was performed with SYBR Green (catalog: 1725124, Bio-Rad, USA) on CFX96 Touch™ Deep Well Real-Time PCR Detection System (Bio-Rad Laboratories, Inc., USA). The parameters used were as follows: 95 ˚C for 3 min, 39 cycles of 95 ˚C for 10 sec, and 60˚C for 30 sec. The assay was performed in triplicate, and data were processed using the 2^-ΔΔCq^ method. Primer sequences are shown in Supplementary Table 4.

### Western blot

Proteins were extracted from tissues harvested at indicated times using radioimmunoprecipitation assay (RIPA) lysis buffer (catalog: R0278, Sigma, USA) supplemented with proteinase inhibitors. Protein concentrations were measured using the BCA Protein assay kit (CW0014S, CWBiotech Co. Ltd., China). Equal amounts of protein were separated on 10% SDS/PAG, followed by immunoblotting with the primary antibodies (anti-proBDNF antibody, 1:500; anti-p75^NTR^, 1:1000; anti-sortilin, 1:1000; anti-p65, 1:1000; anti-RhoA, 1:1000; anti-p-JNK, 1:1000; anti-JNK, 1:1000; anti-GAPDH, 1:5000). Membranes were then incubated with peroxidase-conjugated secondary antibodies, and specific bands were detected with a Bio-Rad imaging system (Hercules, CA).

### PBMCs Isolation

Peripheral blood samples were collected from HDs and MS patients. PBMCs were collected by lymphoprep separation liquid (catalog: 07851, STEMCELL Technologies, Norway) using the standard protocol. The basic medium used for PBMCs is IMDM medium (catalog: 12440-053, Gibco, USA) containing 10% FBS, 1% penicillin-streptomycin solution (catalog: 15140122, Gibco, USA) and 1% L-Glutamine (catalog: 1699722, Gibco, USA). All cells were maintained in a humidified atmosphere of 5% CO_2_ at 37 °C. *In vitro* stimulators include class B CpG oligonucleotide (CpG-B, 3.2 μg/mL), CD40L (1 μg/mL), anti-IgM (10 μg/mL), anti-CD3/CD28 (1:1 ratio), phytohemagglutinin (PHA, 10 μg/mL) and LPS (1 μg/mL) (see Supplementary Table 3).

### Immunohistochemistry

The paraffin-embedded tissue sections were serially cut at 4 μm. Slides were deparaffinized, dehydrated, and retrieved in the citric acid buffer (pH 6.0) in a microwave oven. After cooling, the slides were blocked with normal goat serum and incubated with primary anti-proBDNF (1:200) antibody overnight at 4 °C. The sections were then washed with PBS and incubated with the secondary antibody for 2 h at 37 ℃. The sections were then washed with PBS and stained using the DAB Detection Kit (catalog: ZLI-9017, Zsbio, China). The images were obtained using a microscope (Nikon, Japan) and the percentage of the stained area of proBDNF in human brain sections was analyzed using ImageJ 1.8.0 software as described before 31.

### Immunoprecipitation

Hippocampus or prefrontal cortex tissue lysate from C57BL/6J mice and platelet cell lysates from HDs were incubated with anti-proBDNF antibody (1D3 clone or 2H8 clone, kindly provided by Yile Biology Company) or control IgG and rProtein A/G beads for 4 h. Beads were washed three times for 5 min and the proteins bound to the beads were analyzed by Western blotting with anti-proBDNF antibodies (1:1,000).

### Primary neuron culture

P0-2 pups of C57BL/6J mice were used, and brains were collected in DMEM (catalog: 10569010, Invitrogen, USA) + 1% penicillin-streptomycin on ice. The meninges were removed and the cortex was isolated under a microscope. Four cortices from 2 pups were transferred into a 15 mL tube each containing DMEM + 2% penicillin-streptomycin. The cortices were centrifuged at 1200 rpm 4 °C for 2 min and the supernatant was discarded. One milliliter 0.05% trypsin + 1 mL PBS + 2 μL DNase I (catalog: LS006344 Worthington, USA) per tube were added to digest the cortex for 15 min in a 37 °C incubator. Subsequently, 150 μL FBS was added to deactivate the enzymatic reaction. Digested tissue was dissociated with a fire-polished Pasteur pipette for 10-15 times into small pieces and then left to settle down for a couple of minutes. The supernatant was transferred into a new 15 mL tube and centrifuged to get the cell pellet. The cells were counted and seeded at 5000 cells/cm^2^ in a 12-well plate. The medium was half changed every 2 days. ProBDNF or mono-antibody were added 5 days after seeding. The following four groups were formed: control group without any treatment; proB group with proBDNF; proB+1D3 group with 30 ng/mL proBDNF+3 μg/mL 1D3; proB+2H8 group with 30 ng/mL proBDNF+3 μg/mL 2H8. Cortical neurons were fixed 1 h after treatments. Axon length was analyzed by ImageJ.

### Statistical analysis

All experiments were repeated at least three times, and data were expressed as the mean ± s.e.m. SPSS 18.0 software package (SPSS, Chicago, IL, USA) and GraphPad Prism 7 were used to perform statistical analysis. The difference between the two groups was compared by independent samples t-test. The difference among three or more groups was compared by One-way or Two-way ANOVA with post hoc Bonferroni test. The P-value of less than 0.05 was considered statistically significant.

## Results

### Upregulation of ProBDNF/p75^NTR^ in peripheral immune cells and the CNS in/of MS patients

**Figure 1:**
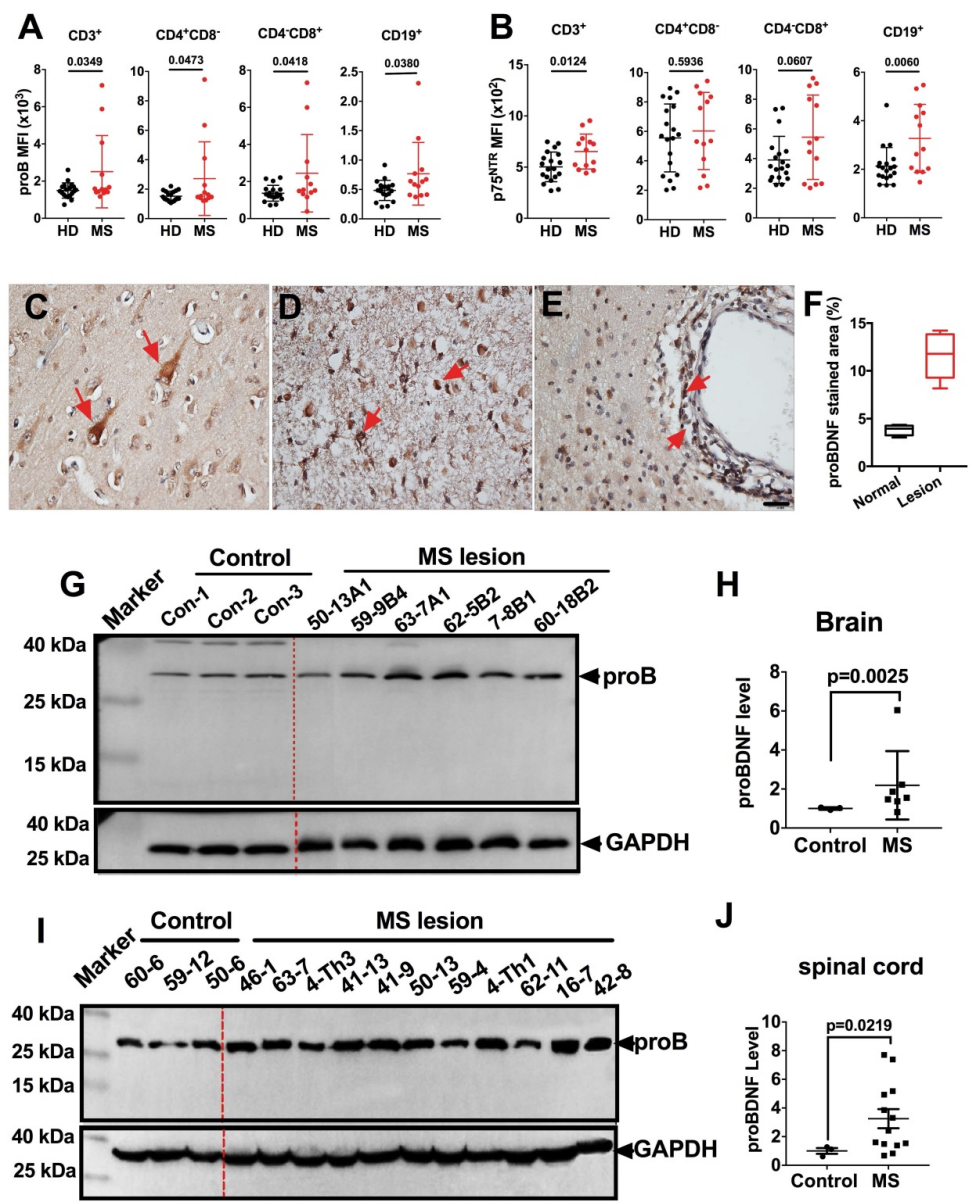
** ProBDNF is upregulated in the blood, brain, and spinal cord of MS patients. (A**-**B)**. PBMCs were isolated from the peripheral blood of HD or MS patients and subjected to flow cytometry analysis. ProBDNF expression was increased in CD3^+^, CD4^+^CD8^-^, CD4^-^CD8^+^ T cells, and CD19^+^ B cells in MS patients compared to HDs (**A**), and p75^NTR^ was increased in CD3^+^ T cells and CD19^+^ B cells (**B**). (**C**-**F**). Representative immunohistochemistry images and statistical analysis showing (**F**) proBDNF positive staining in human brain specimens. ProBDNF was moderately expressed in cortical neurons in the normal brain (**C**, red arrows). ProBDNF was highly expressed in MS patients' brain lesions, particularly around the perivascular area (**D**-**E**, red arrow). Bar = 20 μm. (**G**-**H**). Representative Western blot (**G**) and statistical analysis (**H**) indicating that the proBDNF level significantly increased in MS patients' brain lesions. (**I**-**J**). Increased proBDNF expression in MS post-mortem spinal cord specimens by Western blotting was repeated at least three times (**I**) and statistical analysis (**J**). t-test. Error bars represent mean ± s.e.m. MS: multiple sclerosis.

We first examined the proBDNF expression pattern in peripheral blood PBMCs from MS patients by flow cytometry. ProBDNF expression was increased in all tested immune cells, including CD3^+^, CD4^+^CD8^-^, and CD4^-^CD8^+^ T cells, as well as CD19^+^ B cells, from MS patients compared to healthy donors (Figure 1A and Table S1). Besides, p75^NTR^ was also upregulated in CD3^+^ T cells and CD19^+^ B cells in MS patients (Figure 1B and Table S1). We further studied proBDNF expression in post-mortem brain tissues of MS patients. ProBDNF was mildly expressed in neurons from non-lesion regions of the brain (Figure 1C), but was highly expressed in the infiltrated inflammatory cells at lesion sites, especially in perivascular locations (Figure 1D-F, and Table S2). Compared to controls, proBDNF protein levels were increased in MS brain lesions, as measured by Western blotting (Figure 1G-H, and Table S2). Elevated proBDNF expression was also observed in spinal cord lesions in 9 out of 11 MS patients (Figure 1I-J, and Table S2). These findings suggested that proBDNF upregulation is involved in the pathogenesis of MS.

### ProBDNF is persistently expressed in peripheral immune cells but increased in the CNS only during the late phase of EAE

**Figure 2:**
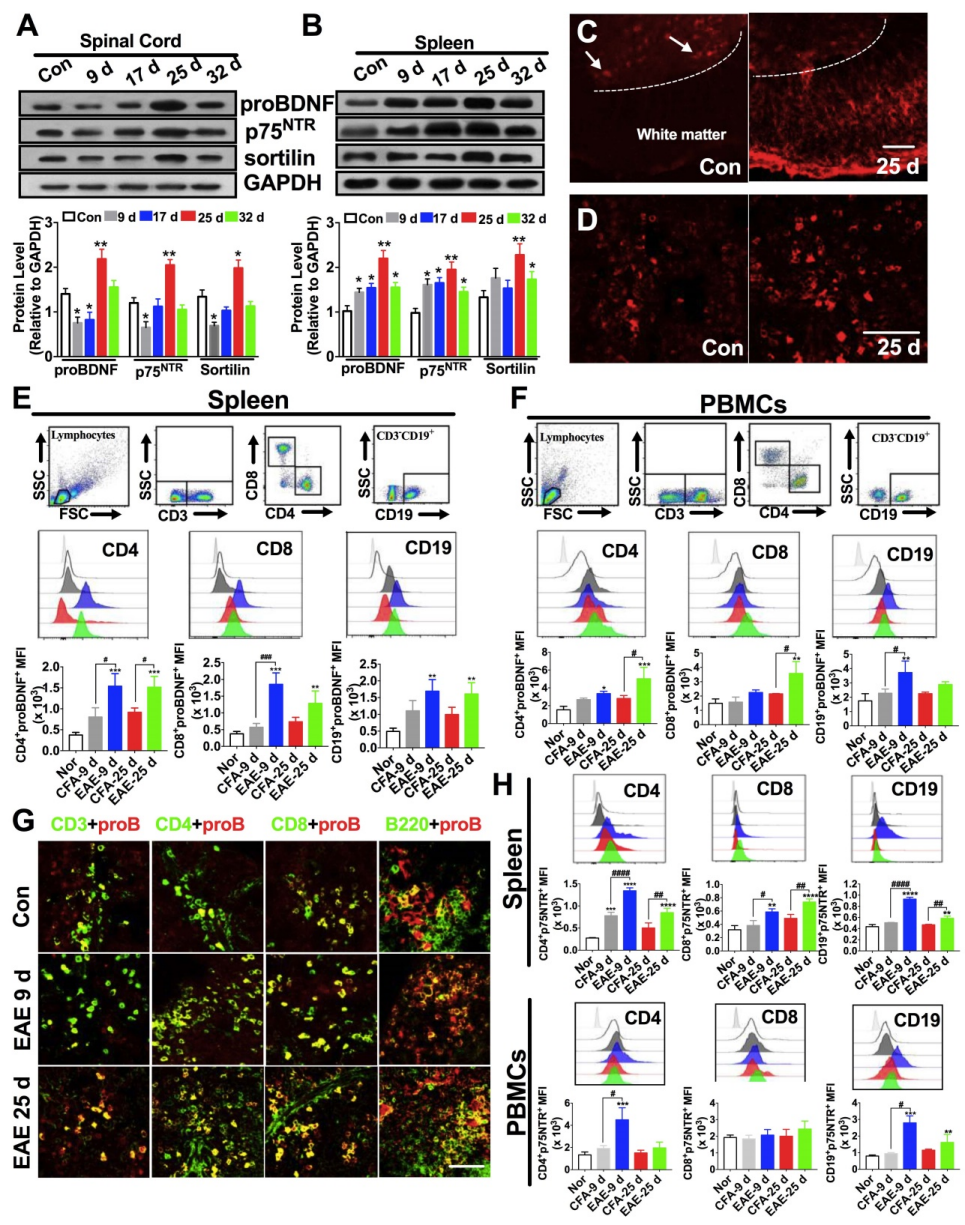
** Expression pattern of proBDNF in the immune system and central nervous system during EAE progression.** C57BL/6J mouse model of EAE was induced by subcutaneous immunization with the completely emulsified MOG35-55/IFA(H37RA) followed by intraperitoneal injection with PTX. The spinal cord, spleen, and peripheral blood were harvested at different time points and tested for proBDNF and p75^NTR^ expression. (**A-B**). Representative Western blot images showing the expression of proBDNF, p75^NTR^, and sortilin in the spinal cord (**A**) and spleen (**B**) during EAE progression. Note that proBDNF and p75^NTR^ were highly expressed in the spleen since the early stage of EAE (9 d), whereas proBDNF expression only increased in the spinal cord at the very late stage (25 d). One-way ANOVA test followed by Bonferroni's multiple comparisons test. *<0.05, **<0.01 compared to controls, n = 6 in each group. (**C-D**). Representative immunofluorescence images of proBDNF positive staining in naïve control and EAE mice at 25 days after immunization in the spinal cord (**C**) and spleen (**D**). Bar = 50 μm (**E**). Splenocytes isolated from EAE mice were stained with cell surface markers CD3, CD4, CD8, and CD19 to test the proBDNF expression by flow cytometry. ProBDNF significantly increased in CD4^+^CD8^-^, CD4^-^CD8^+^, and CD3^-^CD19^+^ cells both at 9 and 25 days after EAE induction compared to naïve mice (Con) or CFA only injected mice (CFA). One-way ANOVA test followed by Bonferroni's multiple comparisons test. **<0.01, ***<0.001 compared to the control, #<0.5, ###<0.001 as indicated, n = 6 in each group. (**F**). PBMCs were isolated from the peripheral blood of EAE mice and tested by flow cytometry. ProBDNF was mainly expressed in CD3^-^CD19^+^B cells at the early stage of EAE (9d), but both increased in CD4^+^CD8^-^ T cells and CD4^-^CD8^+^ T cells at 9 and 25 days after EAE induction compared to naïve mice (Con) and CFA only injected mice (CFA). One-way ANOVA test followed by Bonferroni's multiple comparisons test. *<0.05, **<0.01, ***<0.001 compared to the control, #<0.5, ###<0.001 as indicated, n = 6 in each group. (**G**). Representative immunofluorescence images showing increased proBDNF double staining with CD3, CD4, CD8, and B220 in the spleen at 9 and 25 days after EAE induction compared to naïve mice (Control). Bar = 50 μm (**H**). Flow cytometry showing increased p75^NTR^ expression in CD4^+^CD8^-^ T cells, CD4^-^CD8^+^ T cells, and CD3^-^CD19^+^ B cells in the spleen during EAE development but mainly expressed in PBMCs at the early stage of EAE compared to naïve mice (Con) and CFA only injected mice (CFA). One-way ANOVA test followed by Bonferroni's multiple comparisons test. *<0.05, **<0.01, ***<0.001 compared to the control, #<0.5 as indicated, n = 6 in each group. Error bars represent mean ± s.e.m. Con: naïve mice; CFA: CFA only injected mice; PBMCs: peripheral blood mononuclear cells.

The upregulation of proBDNF in MS patients prompted us to investigate its expression pattern in a mouse model of EAE immunized with the MOG 35-55 peptide. During the peak phase of the disease (25 days after immunization), we confirmed the infiltration of inflammatory cells to the brain and spinal cord (Figure S1A-D) and loss of myelin (Figure S1E-H), as previously reported 30. Western blot analysis showed that proBDNF, p75^NTR^, and sortilin protein levels were significantly decreased in the spinal cord during the initial inductive phase at 9 and 17 days post-immunization, but were significantly increased at 25 days post-immunization in the EAE mice compared to the naïve controls (Figure 2A). In normal mice, immunofluorescence staining for proBDNF in the spinal cord showed that its expression was confined to neuronal cell bodies in the grey matter (Figure 2C). In contrast, in EAE mice, proBDNF was expressed in grey matter neurons and in non-neuronal cells of the white matter (Figure 2C).

Persistent upregulation of proBDNF and its receptors in spleens of EAE mice was detected at all time points examined by Western blot and immunofluorescence (Figure 2B and D). Furthermore, proBDNF was increased in splenic CD4^+^CD8^-^ T cells, CD4^-^CD8^+^ T cells, and CD19^+^ B cells in the early (day 9) and peak (day 25) stages of EAE (Figure 2E). In the PBMCs from EAE mice, the proBDNF level in B cells was increased in the early stage and returned to basal levels at 25 days post-immunization (Figure 2F). However, the proBDNF expression in blood CD4^+^CD8^-^ and CD4^-^CD8^+^ T cells was significantly increased at 25 days post-immunization (Figure 2F). The increased proBDNF expression in splenic T and B cells in EAE mice was further confirmed by confocal microscopy (Figure 2G). Additionally, p75^NTR^ was upregulated in splenocytes and PBMCs in EAE mice (Figure 2H). In the spleen, persistent p75^NTR^ upregulation was detected in CD4^+^CD8^-^ T cells, CD4^-^CD8^+^ T cells, and CD19^+^ B cells (Figure 2H). In PBMCs, however, p75^NTR^ was increased in CD19^+^ B cells and CD4^+^CD8^-^ T cells but not in CD4^-^CD8^+^ T cells (Figure 2H). The increased expression of proBDNF signaling in peripheral lymphocytes and CNS cells in EAE mice supported a possible role of proBDNF in EAE disease progression.

### Peripheral but not CNS administration of Ab-proB neutralizing antibodies attenuates EAE

**Figure 3:**
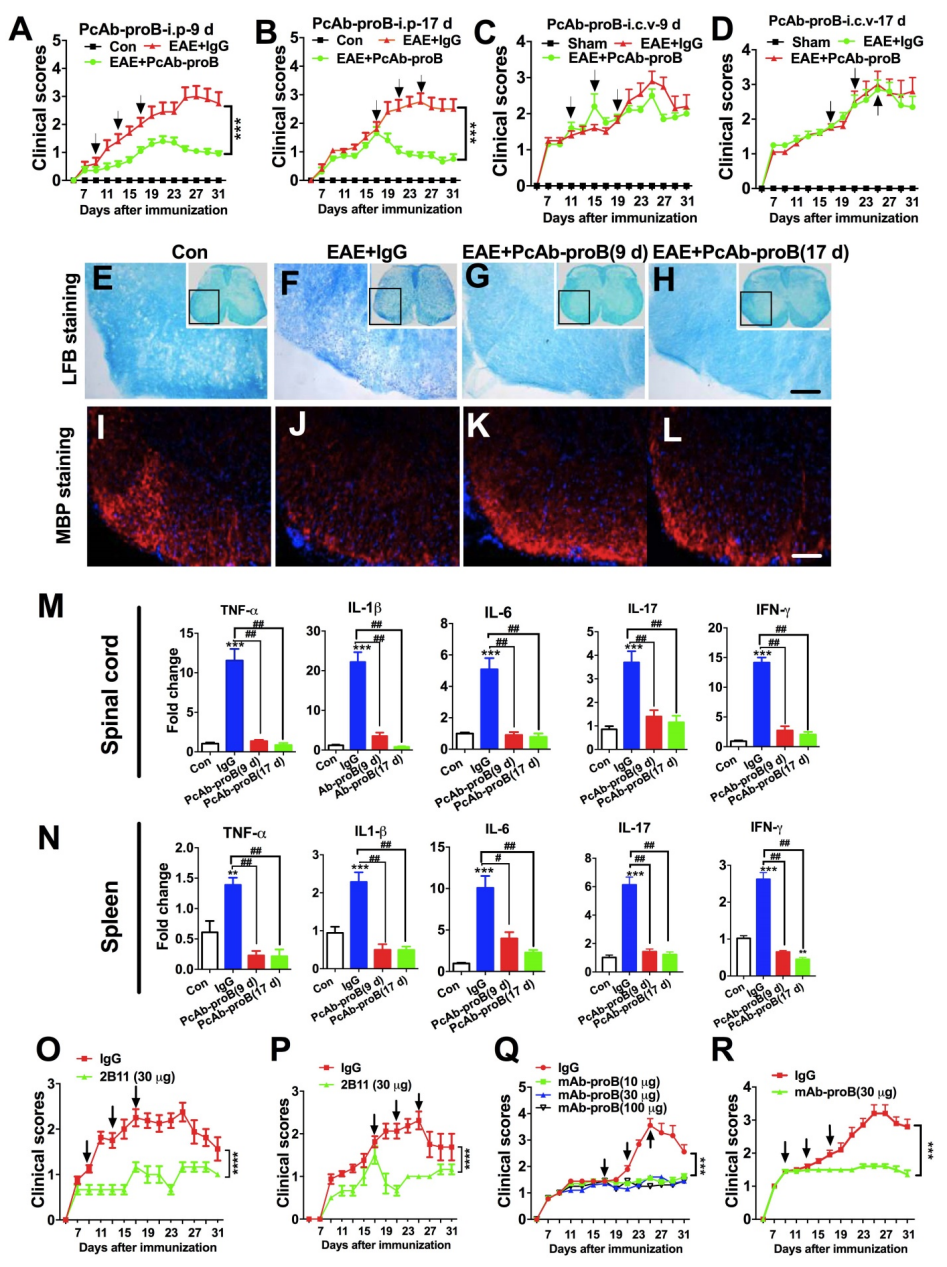
** Protective and inhibitory effects of anti-proBDNF on EAE development.** (**A-B**). EAE mice were *i.p.* injected with PcAb-proB (0.08 μg/μl^.^g) for 3 days at the early stage (**A**, 9, 13, and 17 days) or late stage (**B**, 17, 21, and 25 days) after EAE induction. PcAb-proB significantly reduced clinical scores of EAE mice at both treatment time points. Two-way ANOVA test, ***<0.001, n = 12 in each group. (**C-D**). EAE mice were *i.c.v*. injected with PcAb-proB (1 μg) for 3 days at the early stage (**C**, 9, 13 and 17 days) or late stage (**D**, 17, 21 and 25 days) after EAE induction. The injection of PcAb-proB did not inhibit the clinical symptoms of EAE. (**E-L**). Representative LFB staining (**E-H**) and myelin basic protein (MBP) immunofluorescence images **I-L**) showing obvious myelin loss in the spinal cord of EAE mice (**F** and **J**) compared to naïve mice (**E** and **I**), whereas PcAb-proB significantly reversed the myelin loss both at early (**G** and **K**) and late phase (**H** and **L**) during EAE progression. Each experiment was repeated 3 times, Bar = 50μm. (**M-N**). PcAb-proB *i.p.* injected at 9 or 17 days after EAE induction dramatically suppressed proinflammatory cytokines mRNA levels in the spinal cord (**M**) and spleen (**N**). One-way ANOVA test followed by Bonferroni's multiple comparisons test. *<0.05, **<0.01, ***<0.001 compared to control, #<0.5, ##<0.01, ###<0.001 as indicated, n = 3 in each group, repeated 3 times. (**O-P**). The murine monoclonal anti-proBDNF antibody (2B11) significantly improved clinical scores in both early (**O**, 9, 13, and 17 days) and later (**P**, 17, 21, and 25 days) stages of EAE. (**Q**). Effects of different doses of mAb-proB *i.p.* injections on EAE clinical scores in the late phase of EAE. (**R**). *i.p.* injection of mAb-proB significantly reduced clinical scores of EAE at the early stage after EAE induction. Two-way ANOVA test, ***<0.001, ****<0.0001, n = 12 in each group. Error bars represent mean ± s.e.m. Con: naïve mice; sham: mice treated with *i.c.v*. injection of 1.0 μl saline; PcAb-proB: polyclonal anti-proBDNF; mAb-proB: monoclonal anti-proBDNF; *i.p.*: intraperitoneal; *i.c.v*.: intra-cerebroventricular; MBP: myelin basic protein.

We assessed the role of proBDNF in EAE pathogenesis by administering a sheep polyclonal anti-proBDNF blocking antibody (PcAb-proB) that has been extensively characterized previously 32. Based on the biphasic expression profile of proBDNF in the spinal cord and its consistent expression in the spleen, we tested the time course effect of anti-proBDNF treatment on the EAE model. Injection of PcAb-proB (*i.p.*) at 0.8μg/μl per g of body weight was given at early or late stages after EAE induction, while control mice received the same dose of control IgG. PcAb-proB significantly attenuated the clinical scores two days after the first dose of early or late stage treatment after EAE induction (Figure 3A-B). The improvement of clinical scores continued following the administration of subsequent doses of PcAb-proB (Figure 3A-B). In contrast, the clinical scores in the control mice deteriorated over time (Figure 3A-B). To test whether PcAb-proB's therapeutic effect was due to local inhibition of proBDNF in the CNS, we administered 1 μg of PcAb-proB or control IgG to EAE mice with three *i.c.v*. injections at 9 days and 17 days post-induction (Figure 3C-D). We found that *i.c.v.* administration of PcAb-proB did not improve clinical scores (Figure 3C-D), indicating that the therapeutic effect of PcAb-proB was not due to its effects on the CNS, but on the peripheral system.

Compared with the control IgG-treated mice, peripheral administration of PcAb-proB starting at either 9 days or 17 days post-immunization attenuated the loss of myelin (Figure 3E-H) and restored myelin basic protein (MBP) expression (Figure 3I-L). Notably, *i.p.* PcAb-proB administration decreased the gene expression of proinflammatory cytokines, including tumor necrosis factor (TNF)-α, interleukin (IL)-1β, IL-6, IL-17, and interferon (IFN)-γ in the spinal cord (Figure 3M) and spleen (Figure 3N). These results indicated that the therapeutic effects of PcAb-proB might be partly mediated by the immune system.

Furthermore, the administration of a mouse anti-proBDNF monoclonal antibody (clone 2B11) 22 improved the clinical scores of EAE mice (Fig 3O and 3P). Finally, we developed a humanized monoclonal Ab-proB (mAb-proB) and analyzed its translational potential. The humanized mAb-proB neutralized proBDNF, as determined by immunoprecipitation and neurite growth assays (Figure S2). Importantly, mAb-proB i.p. injection significantly reduced clinical scores in EAE mice when administered at late or early stages after EAE immunization (Fig 3Q and R), suggesting that mAb-proB conferred protection against disease development in the EAE mouse model. In the EAE mice immunized with MOG 35-55 and IFA (H37RA 4 mg/mL), myelin-specific T cells are essential for initiating CNS inflammation and clinical manifestations 33. In contrast, immunization with rhMOG induces B cell-dependent EAE mice with hallmarks of B cell activation and anti-MOG antibody production 34. Treatment with *i.p.* mAb-proB ameliorated the disease progress in EAE mice induced with rhMOG immunization (Figure S3). Thus, these results showed that mAb-proB conferred protection in different EAE models.

### Upregulation of proBDNF and p75^NTR^ in the immune cells upon immune stimulation

**Figure 4:**
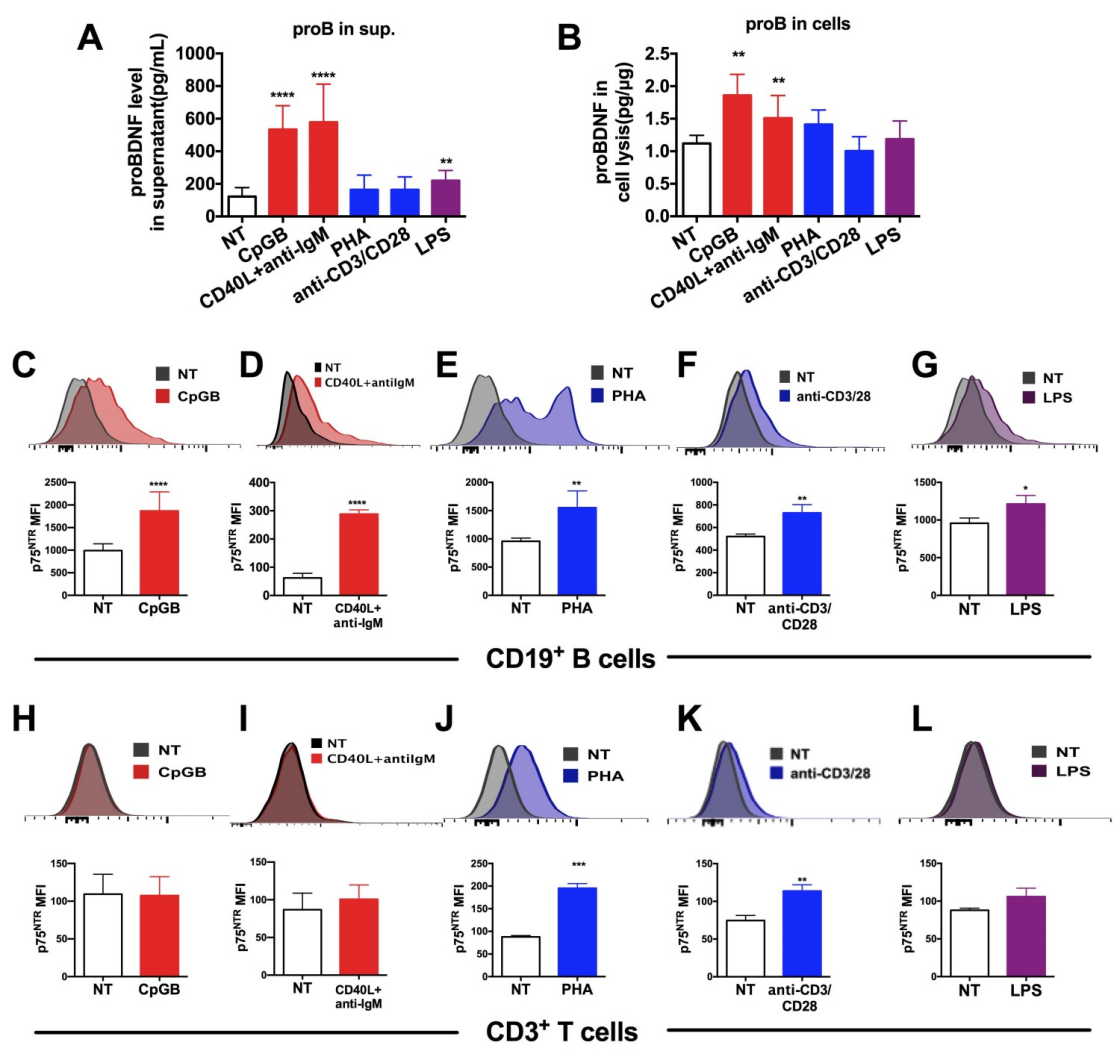
** Upregulation of proBDNF and its receptor p75^NTR^ in human lymphocytes following treatment with various stimulants.** (**A-B**). PBMCs were isolated from the peripheral blood of HDs and subjected to various stimulations for 3 days. Supernatant and cells were collected and used to test proBDNF levels by ELISA. B cell stimulants CpG-B or CD40L+anti-IgM treatment increased the proBDNF level significantly in PBMCs (red bars) both in the supernatant (**A**) and in cells (**B**), but the stimulants that preferentially activated T cells, including PHA and anti-CD3/CD28 (blue bars) did not increase proBDNF level. LPS upregulated proBDNF level in the supernatant but not in cells. One-way ANOVA test followed by Bonferroni's multiple comparisons test. **<0.01, ****<0.0001 compared to NT, n = 4 in each group, repeated at least 3 times. (**C-G**). HD PBMCs were isolated and subjected to various stimulations for 3 days. Representative flow cytometry images (upper panel) and statistical analysis (lower panel) indicating CpG-B, CD40L+anti-IgM, PHA, anti-CD3/CD28, and LPS increased the expression of membrane p75^NTR^ in CD19^+^ B cells in HD PBMCs. (**H-L**) Treatment with T cells activators PHA or anti-CD3/CD28 dramatically increased p75^NTR^ in CD3^+^ T cells in activated HD PBMCs, but treatment with CpG-B, CD40L+anti-IgM, or LPS did not affect p75^NTR^ level. t-test, *<0.5, **<0.01, ***<0.001, ****<0.0001, n = 3 in each group, repeated at least 3 times. Error bars represent mean ± s.e.m. NT: no treatment; sup.: supernatant.

In an attempt to investigate whether proBDNF signaling contributed to EAE disease progression by regulating immune function, we measured the *in vitro* expression of proBDNF and p75^NTR^ in isolated human PBMCs following stimulation. Human PBMCs were cultured in various conditions known to stimulate innate and adaptive immune responses via B cells, T cells, and monocytes. The concentration of proBDNF in the supernatant and cell lysates was then measured and the surface expression of p75^NTR^ in lymphocytes was analyzed (Figure 4).

Results showed that the proBDNF concentration in the supernatant was significantly increased following PBMCs stimulation with the Toll-like receptor 9 (TLR9) ligand CpG-B, the costimulatory molecule CD40 ligand (CD40L), and anti-IgM antibodies (for B cells antigen receptor (BCR) stimulation), all of which are known to activate B cells preferentially (Figure 4A). Besides, a moderate but significant increase in proBDNF concentration was observed after LPS stimulation of PBMCs (Figure 4A). ELISA analysis of whole PBMCs lysates showed that intracellular proBDNF was significantly increased upon CpG-B treatment and CD40L+anti-IgM stimulation (Figure 4B). Flow cytometry also revealed that the increase in proBDNF following CpG-B and CD40L + anti-IgM stimulation was confined to CD19^+^ B cells in PBMCs (Figure S4). Moreover, p75^NTR^ expression was significantly increased in B cells after activation of PBMCs by CpG-B and CD40L+anti-IgM, and T cell stimulants, including PHA and anti-CD3/28 (Fig. 4C-G). P75^NTR^ expression also increased in T cells after activation of PBMCs by PHA and anti-CD3/28 beads (Figure. 4H-L). These findings suggested that proBDNF signaling in the immune system is involved in neuroinflammation during EAE/MS development.

**Figure 5:**
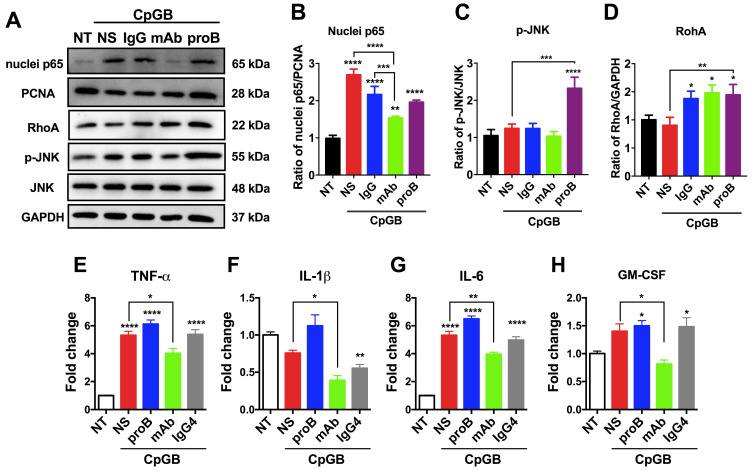
** ProBDNF promotes proinflammatory cytokine expression of lymphocytes from HDs in vitro.** (**A-D**) PBMCs were isolated from HDs and cultured in the presence of CpG-B (3.2 μg/mL) with or without (NS) recombinant proBDNF protein (100 ng/mL) or mAb-proB (2 μg/mL) for 3 days. Cells were harvested, and cell lysates were used for Western blotting. Representative images of Western blots (**A**) and statistical analysis (**B-D**) of NF-κB (nuclear and cellular p65), RhoA, and p-JNK. One-way ANOVA followed by Bonferroni's *post-hoc* pairwise comparisons (*p<0.05, **p<0.01, ***p<0.001, ****p<0.0001 compared to NT, n = 3 in each group, repeated 3 times). (**E-H**). HD PBMCs were treated with CpG-B for 24 h, and the effect of exogenous proBDNF protein (100 ng/mL), mAb (2 μg/mL) or isotype control IgG4 (2 μg/mL) on proinflammatory cytokine expression was evaluated. QPCR of PBMCs showed that mAb inhibited the upregulation of TNF-α, IL-1β, IL-6, and GM-CSF in PBMCs induced by treatment with CpG-B. One-way ANOVA test followed by Bonferroni's multiple comparisons test. *<0.05, **<0.01, ****<0.0001 compared to NT or as indicated, n = 3 in each group, repeated at least 3 times. Error bars represent mean ± s.e.m. NT: no treatment; NS: saline; proB: proBDNF protein; mAb: monoclonal anti-proBDNF antibody.

Next, we examined the downstream signaling of proBDNF/p75^NTR^ in the immune system upon the immune stimulant challenge. It has been reported that in CNS, the downstream signaling of p75^NTR^ includes NF-κB, RhoA, and JNK 35, 36. We observed that CpG-B treatment activated p65 in PBMCs. Blocking proBDNF with mAb-proB significantly inhibited CpG-B-mediated activation of p65 (Figure 5A-D), and exogenously added proBDNF significantly increased CpG-B-mediated activation of JNK and RhoA (Figure 5A). However, CpG-B treatment did not activate JNK or RhoA pathways. These findings suggested that proBDNF/p75^NTR^/NF-κB signaling axis plays a critical role in the immune response of lymphocytes to CpG-B stimulation. Given that NF-κB mediated various proinflammatory gene expression, we examined whether mAb-proB inhibited the activation of proinflammatory cytokine gene expression after CpG-B treatment. As shown in Figure 5E-H, mAb-proB significantly inhibited the expression of proinflammatory cytokines, including TNF-⍺, IL-1β, IL-6, and GM-CSF in CpG-B-stimulated PBMCs.

### Systemic administration of mAb-proB normalizes the expression of T and B cell population in EAE mice

**Figure 6:**
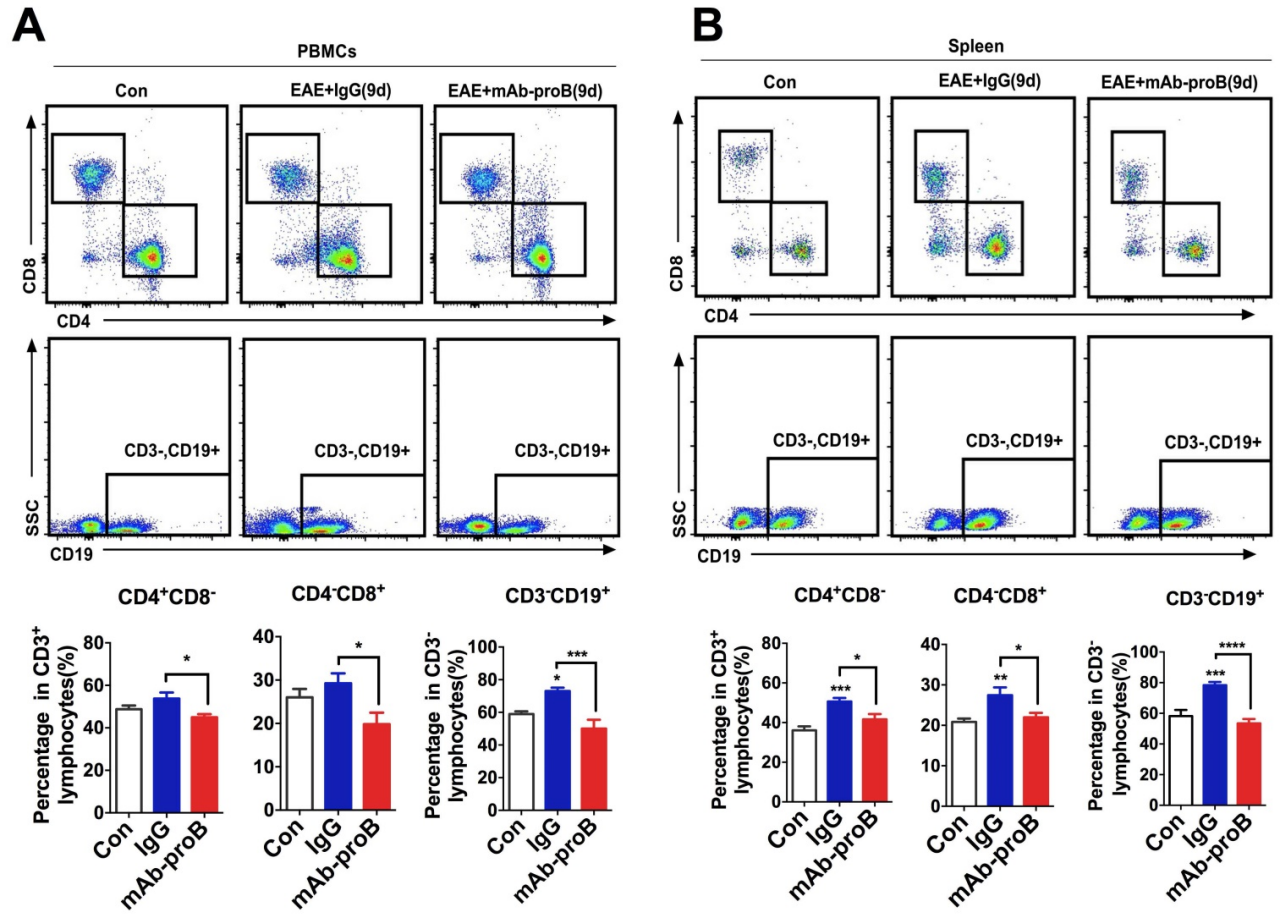
** Monoclonal anti-proBDNF antibody pretreatment greatly inhibits the production of immune cells in EAE mice.** (**A-B**). C57B/L6J mice were i.p. injected with mAb-proB (100 μg) and EAE was induced by subcutaneous injection with completely emulsified MOG35-55/IFA(H37RA) followed by intraperitoneal injection with PTX. PBMCs from peripheral blood and spleen were collected at 9 days after EAE induction and tested by flow cytometry. Representative flow cytometry images (upper panel) and statistical analysis (lower panel) showing the increased percentage of CD4^+^CD8^-^ T cells, CD4^-^CD8^+^ T cells, and CD3^-^CD19^+^ B cells in PBMCs and spleen of EAE mice, whereas mAb-proB pretreatment dramatically blocked these effects in PBMCs (**a**) and spleen (**b**). One-way ANOVA test followed by Bonferroni's multiple comparisons test. *<0.05, ***<0.001, ****<0.0001 compared to control or as indicated, n = 6 in each group. Error bars represent mean ± s.e.m. Control: naïve mice; mAb-proB: monoclonal anti-proBDNF antibody; PBMCs: peripheral blood mononuclear cells.

Our recent studies have shown that proBDNF signaling perturbs immune cell function in the aortic dissection disease and septic mice 23, 25. The therapeutic effect of blocking endogenous proBDNF may be due to normalizing immune cell dysfunction in EAE mice. To test this hypothesis, we explored the effect of mAb-proB on lymphocyte subpopulation levels following EAE induction. MAb-proB pretreatment decreased the levels of CD3^-^CD19^+^ B cells, CD4^+^CD8^-^ T cells, and CD4^-^CD8^+^ T cells, both in the spleen and circulation of EAE mice (Figure 6).

## Discussion

Several important findings emerged from the present study. First, we observed upregulation of proBDNF and its receptors in immune cells of both MS patients and EAE mice, including blood, post-mortem brains, and spinal cords. Second, we defined a detrimental role of proBDNF in the pathogenesis of demyelination and CNS inflammation in EAE mice and affirmed that proBDNF is a therapeutic target to ameliorate disease progression. Third, we discovered that proBDNF/p75^NTR^/NF-κB signaling is implicated in the immune cell dysfunction in the EAE disease. Hence, we propose that suppression of proBDNF biological functions is a promising strategy to treat MS by modulating the perturbed immune system functions.

BDNF, a neurotrophic factor widely expressed in the CNS, is believed to ameliorate the EAE disease course and promote neuronal survival, differentiation, and synaptic plasticity by activating its high-affinity receptor tyrosine receptor kinase (Trk) B. However, in the acute phase of the EAE disease, BDNF gene expression in the spinal cord was downregulated 11. Our study showed that proBDNF was also decreased in CNS in the acute phase, but was upregulated in the late phase of EAE. Given that proBDNF mainly exerts its function by activating p75^NTR^ and negatively regulating synaptic plasticity and axonal growth, the blocking antibody would be effective by neutralizing the upregulated endogenous proBDNF in EAE. We detected that proBDNF blocking antibodies exerted their preventive and therapeutic effects on neuronal and immunomodulatory functions. This contention was supported by the observation that proBDNF signaling was persistently increased in the spleen and circulating T cells and B cells in the EAE mice. Treatment with Ab-proB largely abolished cytokine activation in the spleen and spinal cord, accompanied by improvement in clinical outcomes. Consistently, our recent studies also showed upregulation of proBDNF/p75^NTR^ signaling in the T lymphocytes and monocytes from the patients with sepsis and aorta dissection disease 23, 25. We also observed high expression of proBDNF in the active immune cells in the blood and brain tissues of MS patients, highlighting proBDNF's role in immune cells during MS progression.

Mature BDNF in immune cells plays a neuroprotective role in EAE as its overexpression in T cells has been shown to inhibit axonal damage 37. In the current study, both PcAb-proB and mAb-proB greatly improved the clinical disease score and reduced demyelination and proinflammatory cytokine activation. The therapeutic effects of proBDNF blocking antibodies may be due to neutralizing axonal pruning by proBDNF. However, intracerebroventricular (*i.c.v*.) injection of Ab-proB did not attenuate disease progression, suggesting that the protective effect of Ab-proB was less likely through the direct effect on the CNS. Instead, the axonal protection by systemic Ab-proB was likely secondary to the improvement of immune functions. As T and B cells upregulate proBDNF-p75^NTR^ signaling after antigen challenge, proBDNF may play proinflammatory functions via an autocrine and/or paracrine mechanism. Indeed, mAb-proB inhibited the proinflammatory cytokine gene expression *in vitro* and reduced the number of both T and B cells in EAE mice (Figure 6).

**Figure 7:**
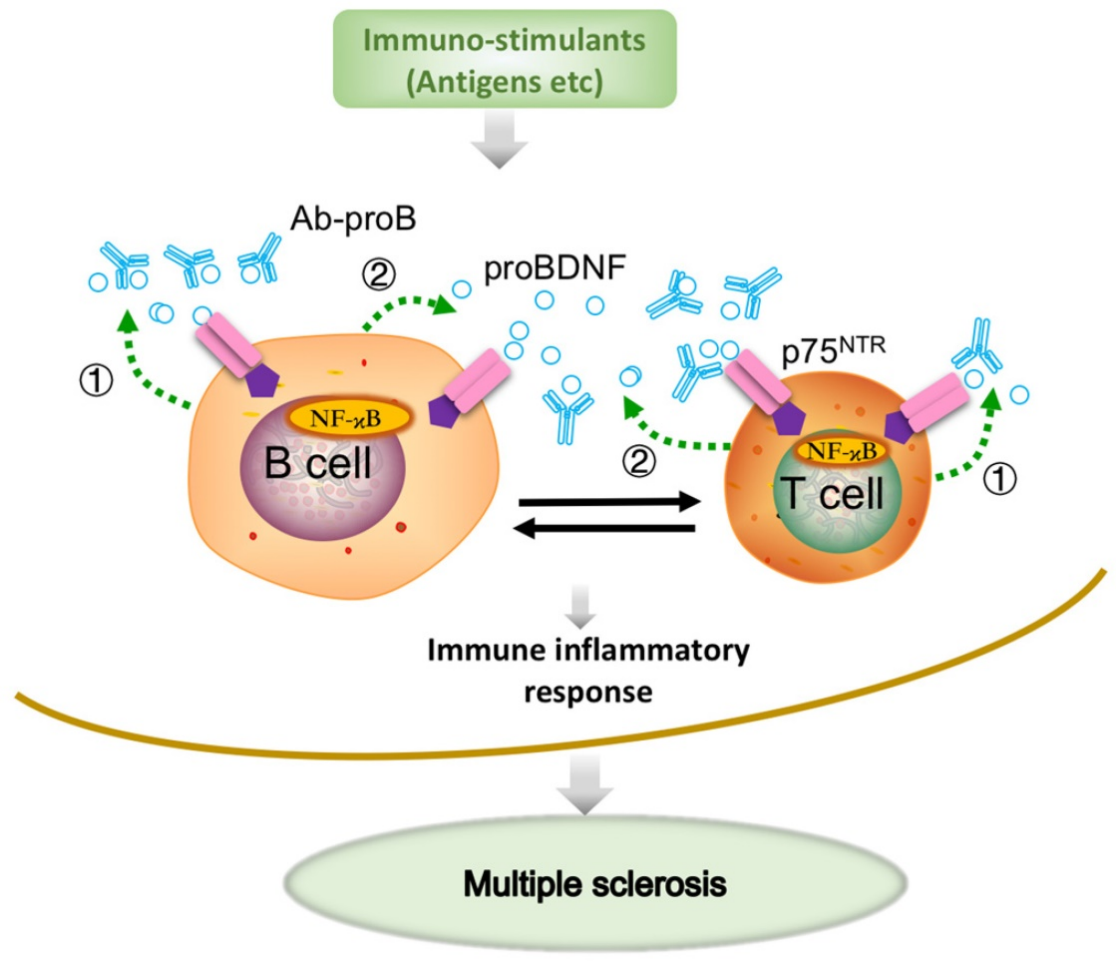
** Schematic diagram shows that Ab-proB ameliorates multiple sclerosis in the EAE mouse model by suppressing proinflammatory immune activity of lymphocytes.** The expression of proBDNF and its receptor p75NTR increases in B cells and other immune cells when challenged with MOG antigen or other immuno-stimulants. The lymphocyte-released proBDNF acts on lymphocytes and/or other immune cells, leading to multiple sclerosis (①). The released proBDNF binds to its high-affinity receptor p75NTR highly expressed on lymphocytes as an autocrine factor (①) as well as a paracrine factor (②). The released proBDNF facilitates inflammatory immune response and blocking with anti-proBDNF antibody disrupts the positive feedback (③), suppressing immune activity in lymphocytes and producing therapeutic effects for multiple sclerosis.

Previous studies have demonstrated the upregulation of proBDNF and its receptor p75^NTR^ in macrophages, monocytes, and microglia in a variety of neurological conditions, including spinal cord injury 21 and *Toxoplasma* infection 20. A more recent study showed that the upregulated p75^NTR^ was mainly expressed in the anti-inflammatory, but not proinflammatory M1 phenotype microglia in the spinal cord during EAE progression 38. We have recently demonstrated that proBDNF was preferentially expressed in M2-like monocytes in acute aortic dissection patients 23. The preferential expression of proBDNF/p75^NTR^ in M2 macrophages/microglia suggested that i.c.v. blockage of proBDNF/p75^NTR^ signaling could not inhibit the proinflammatory effect in CNS in EAE. Thus, our results implied that Ab-proB exerts its protective effects mainly through peripheral immune cells but not via CNS mechanisms. In this scenario, the local release of proBDNF from immune cells modulates the immune cell function and contributes to the EAE pathogenesis (Figure 7).

BDNF and proBDNF can be released from B cells and T cells, contributing to B cell development and T cell maturation 19, 39. ProBDNF was reported to be released from B cells and likely involved in B cell survival 17. A remarkable finding in our present study showed that proBDNF was predominantly released by B cell stimulants. In contrast, the high-affinity receptor p75^NTR^ was upregulated by both T and B cell stimulants, suggesting that the proBDNF/p75^NTR^ signaling may mediate various immune functions. The downstream effectors of p75^NTR^ signaling were reported to include RhoA 16, JNK 36, and NF-κB 35 pathways. Also, GpG-B could activate NF-κB, essential for B cell development, proliferation, and differentiation into plasma cells 17, 19, 27. The decrease in NF-κB activation by mAb-proB suggested that proBDNF plays a role in CpG-B-mediated activation of the NF-κB pathway. Taken together, proBDNF released from active immune cells may exert its biologic effect on B cells and T cells through autocrine and paracrine effects in EAE pathogenesis (Figure 7).

We found that the therapeutic effect of Ab-proB on EAE largely depended on blocking the p75^NTR^/NF-κB pathway, as indicated by our *in vitro* results. However, a previous study reported that p75Exon IV null mutation exacerbated the disease and increased T cell infiltration into the CNS 40. This discrepancy in the effects on the EAE disease course after blocking p75^NTR^ functions might be due to the different strategies of blocking p75^NTR^ in our study versus the previous study. Knocking out exon IV of the *p75* gene would generate a truncated extracellular isoform of the receptor, and the remaining intracellular p75^NTR^ isoform may have pro-apoptotic effects and exacerbate EAE 41. Further studies are needed to investigate the role of p75^NTR^ in the EAE pathogenesis either by using conditional knockout mice or ablation of different exons.

The increased proBDNF expression in immune cells in the animal model and MS patients in Figure 1 and 2 suggests that blocking increased endogenous proBDNF represents a novel therapeutic strategy for treating MS. Indeed, antibodies specifically targeting proBDNF without affecting mature BDNF attenuated the disease severity when administered following EAE induction. Thus, mAb-proB is a promising drug candidate for the clinical application in MS. The underlying mechanism of how proBDNF regulates the EAE disease course is not fully understood because the genetic manipulation of proBDNF would also affect BDNF expression, confounding the phenotype of the EAE disease model. However, the immune cell modulatory function of mAb-proB might have a potential advantage compared with the biological agents, such as anti-CD20 antibodies 42, 43, depleting the immune cells, since the long term B cell depletion would cause potential infections and/or other side effects 44.

In conclusion, our study has reported a novel regulatory function of proBDNF in immune cells alleviating the MS pathogenesis. The humanized mAb-proB may effectively attenuate disease progression and represents a promising therapeutic strategy for treating human MS.

## Supplementary Material

Supplementary figures and tables.

## Abbreviations

ProBDNF: brain-derived neurotrophic factor precursor
CNS: central nervous system
MS: multiple sclerosis
EAE: experimental autoimmune encephalomyelitis
PBMCs: peripheral blood mononuclear cells
P75^NTR^: p75 pan-neurotrophic receptors
BCDT: B cells depletion therapy
Ab-proB: anti-proBDNF blocking antibodies
LPS: Lipopolysaccharide
i.p.: Intraperitoneally
PTX: pertussis toxin
MOG: myelin oligodendrocyte glycoprotein
rhMOG: recombinant human MOG
Mtb: mycobacterium tuberculosis
CFA: complete Freund's adjuvant
PcAb-proB: sheep polyclonal anti-proBDNF blocking antibody
mAb-proB: monoclonal anti-proBDNF blocking antibody
LFB: Luxol Fast Blue
MBP: myelin basic protein
HDs: healthy donors
TLR9: Toll-like receptor 9
CpG-B: class B CpG oligonucleotide
PHA: Phytohemagglutinin
i.c.v.: Intracerebroventricular
