# Supplementary material for: Brain-derived neurotrophic factor precursor in the immune system is a novel target for treating multiple sclerosis
Source: Theranostics. 2021 Jan 1;11(2):715–30. doi: 10.7150/thno.51390 (PMC7738849; doi:10.7150/thno.51390)
Supplement: Supplementary file 1 — Supplementary figures and tables. [file thnov11p0715s1.pdf]

Brain-derived neurotrophic factor precursor in the immune system is a novel target for treating multiple sclerosis

Zhao-Lan Hu[1,2*], Cong Luo[1*], Plinio Reinaldo Hurtado[3*], Hui Li[1], Shuang Wang[5], Bo Hu[6], Jun-Mei Xu[1], Yang Liu[4,7], Shi-Qing Feng[7], Kang Chen[8], Xin-Fu Zhou[4#], Chang-Qi Li[2#] and Ru-Ping Dai[1#]

Affiliations:

1. Department of Anesthesiology, The Second Xiangya Hospital, Central South University, 139 Ren-Min Central Road, Changsha City, Hunan 410011, China.

2. Department of Anatomy and Neurobiology, School of Basic Medical Science, Central South University, Changsha City, Hunan 410011, China.

3. Department of Renal Medicine, Royal Adelaide Hospital, Adelaide, SA 5000, Australia.

4. School of Pharmacy and Medical Sciences, Division of Health Sciences, University of South Australia, Adelaide, SA 5000, Australia.

5. Department of Medical Research Center and Clinical Laboratory, Xiangya Hospital, Central South University, Changsha, Hunan 410008, China.

6. Department of neurology, Xiangya Hospital of Central South University, Changsha, Hunan 410008, China.

7. Department of Orthopedics, Tianjin General Hospital, Tianjin Medical University, Tianjin 300070, China.

8. Perinatology Research Branch, *Eunice Kennedy Shriver* NICHD, National Institutes of Health, Department of Obstetrics and Gynecology, Wayne State University, Detroit, Michigan 48201, USA.

* These authors contribute equally; # senior authors contribute equally.

Corresponding author:

Ru-Ping Dai, MD, PhD,

Department of Anesthesiology, The Second Xiangya Hospital, Central South University, 139 Ren-Min Central Road, Changsha City, Hunan 410011, China. Email: xyeyyrupingdai@csu.edu.cn

Or Chang-Qi Li, PhD,

Department of Anatomy and Neurobiology, School of Basic Medical Science, Central South University, Changsha City, Hunan 410011, China. Email: changqili@csu.edu.cn

Or Xin-Fu Zhou, PhD,

School of Pharmacy and Medical Sciences, Division of Health Sciences, University of South Australia, Adelaide, SA 5000, Australia. Email: Xin-fu.zhou@unisa.edu.au

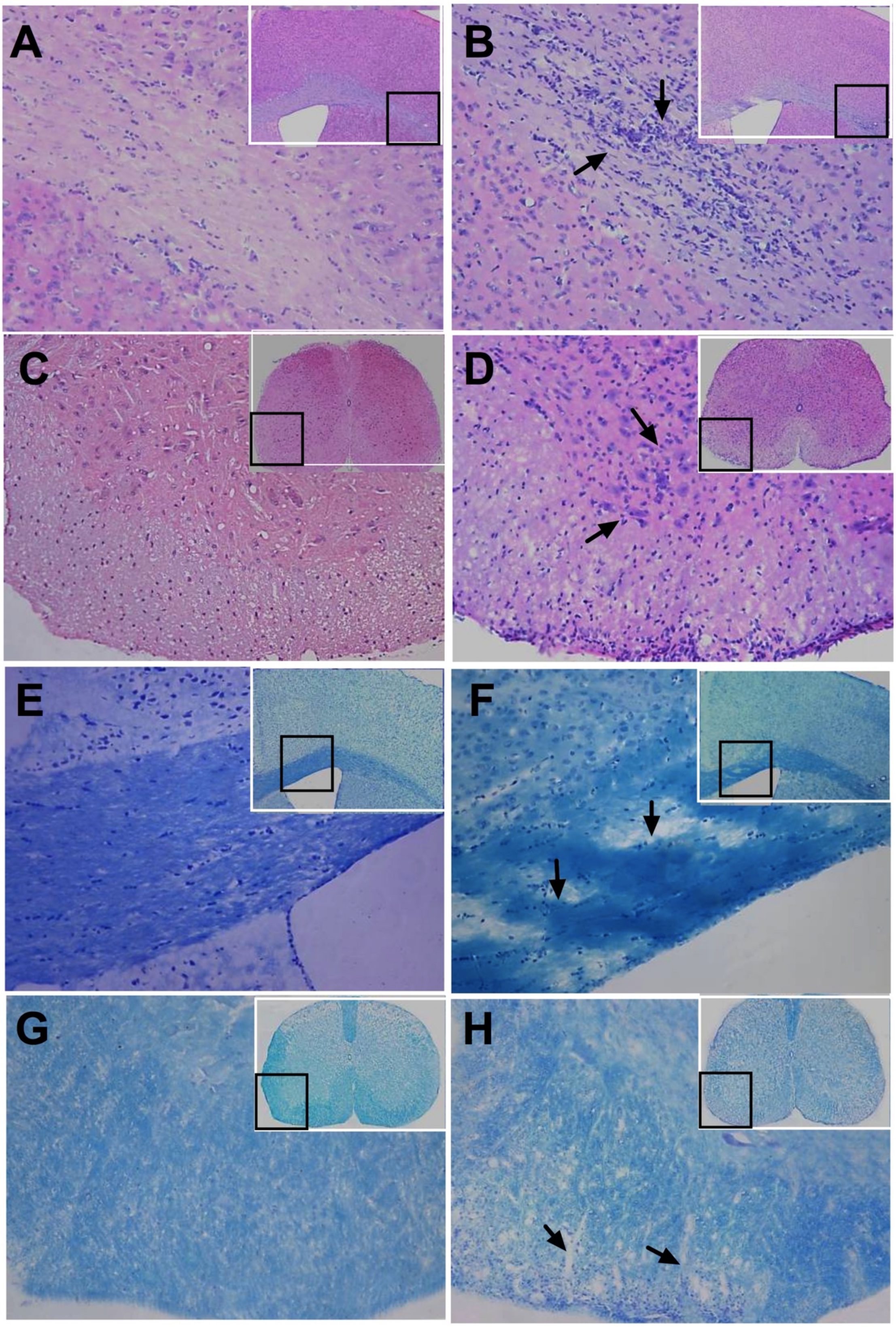

Supplemental Figure 1. High infiltration of inflammatory cells and myelin loss in central nervous system of EAE mice. EAE C57BL/6J mouse model was induced by

subcutaneous injection with completely emulsified MOG35-55/IFA(H37RA) followed by intraperitoneal injection with PTX, brain and spinal cord specimen were harvested at 25 days after injection. (**A–D**) A representative H&E staining showed that numerous inflammatory cells (dark narrow) infiltrated the corpus callosum (**B**) and spinal cord (**D**) compared to naïve control mice (**A** and **C**). (**E–H**) Significant loss of myelin (dark narrow) in brain (**F**) and spinal cord (**H**) in EAE mice compared to naïve control mice (**G** and **H**) as indicated by LFB staining. n>3 in each group, repeated 3 times.

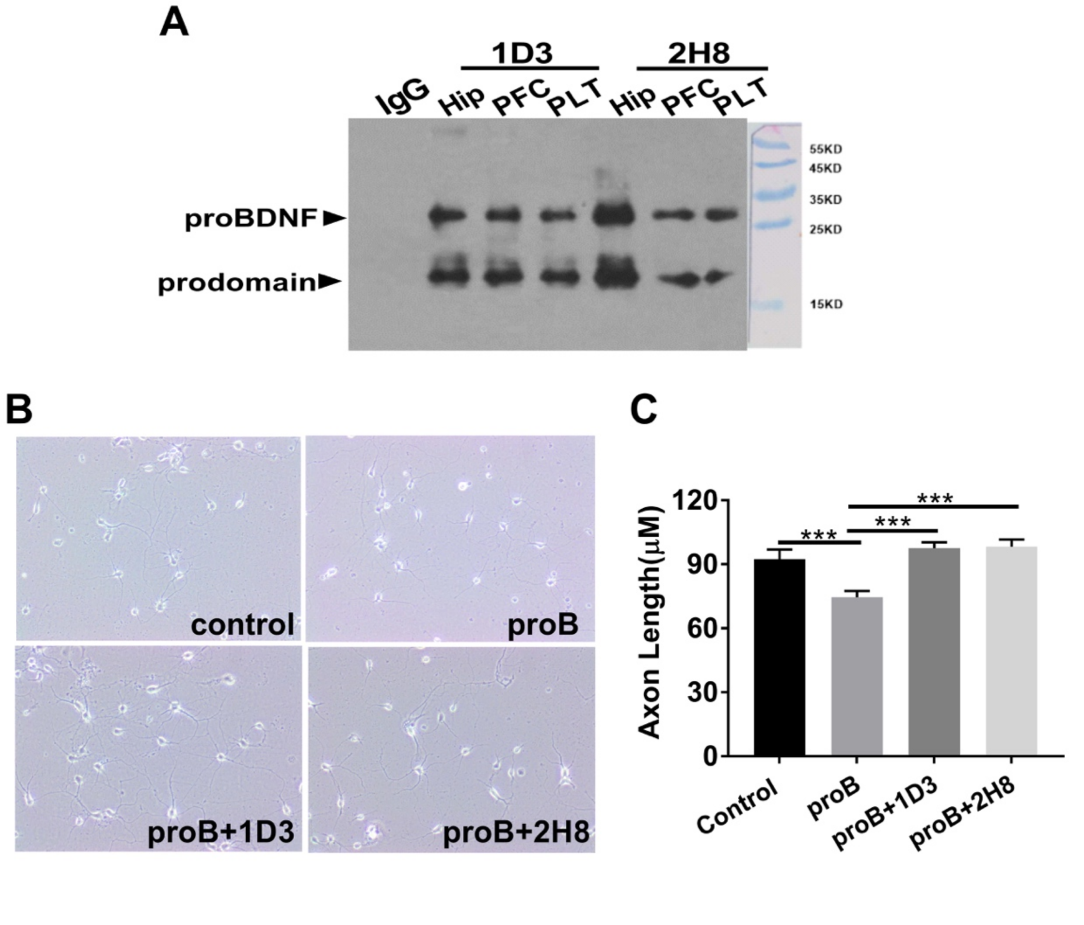

Supplemental Figure 2. Characterization of specificity and biological function of monoclonal anti-proBDNF antibody. (**A**) Representative images showing different monoclonal anti-proBDNF antibody clones including 1D3 and 2H8 clones which

specifically recognized full length proBDNF and its prodomain part in hippocampus (Hip), prefrontal cortex (PFC) and platelet (PLT) of mice. (**C** and **C**) Representative photographs of neonatal neurons and corresponding statistical analysis showing that 1D3 and 2H8 clones specifically blocked the biological function of proBDNF. Neonatal neurons were cultured *in vitro* until they formed dendrites for 7 days and then treated with proBDNF protein in the presence or absence of 1D3 or 2H8 for 24 h. Note that exogenous proBDNF protein dramatically suppressed neuritis outgrowth, whereas 1D3 and 2H8 inhibited this effect. One-way ANOVA followed by Bonferroni's multiple comparison post hoc test, ***p<0.001 as indicated, n = 4 in each group, tested in at least 3 independent experiments. Error bars represent mean ± s.e.m. Hip: hippocampus; PFC: prefrontal cortex; PLT: platelet; 1D3 and 2H8: different clones of monoclonal anti-proBDNF antibody.

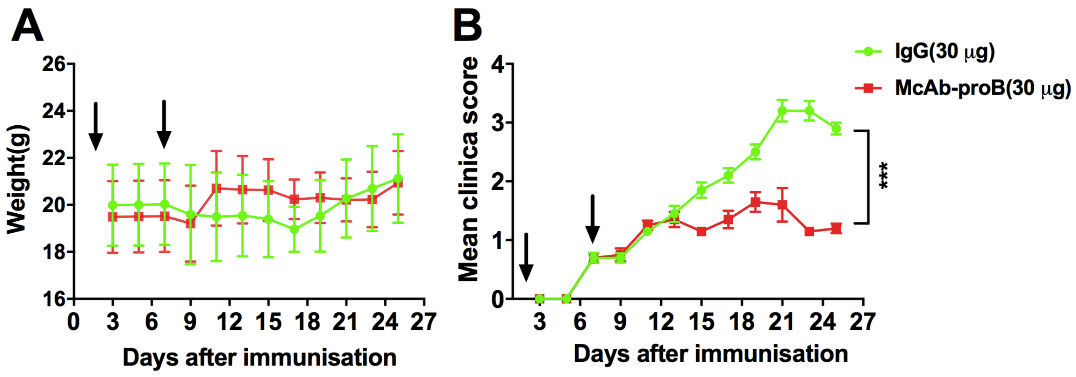

Supplemental Figure 3. Monoclonal anti-proBDNF antibody reduces clinical scores of rhMOG in EAE mice. EAE C57BL/6J mouse was established by subcutaneous injection with completely emulsified rhMOG/CFA followed by intraperitoneal injection with PTX. (**A**) No significant changes in weight were observed between mice treated with IgG control or monoclonal anti-proBDNF antibody (mAb-proB). (**B**) MAb-proB greatly ameliorated clinical manifestation of EAE (0, 7 days) mice after treatment with rhMOG compared to IgG. Two-way ANOVA test,

***p<0.001 as indicated, n = 12 in each group. Error bars represent mean ± s.e.m. MAb-proB: monoclonal anti-proBDNF antibody.

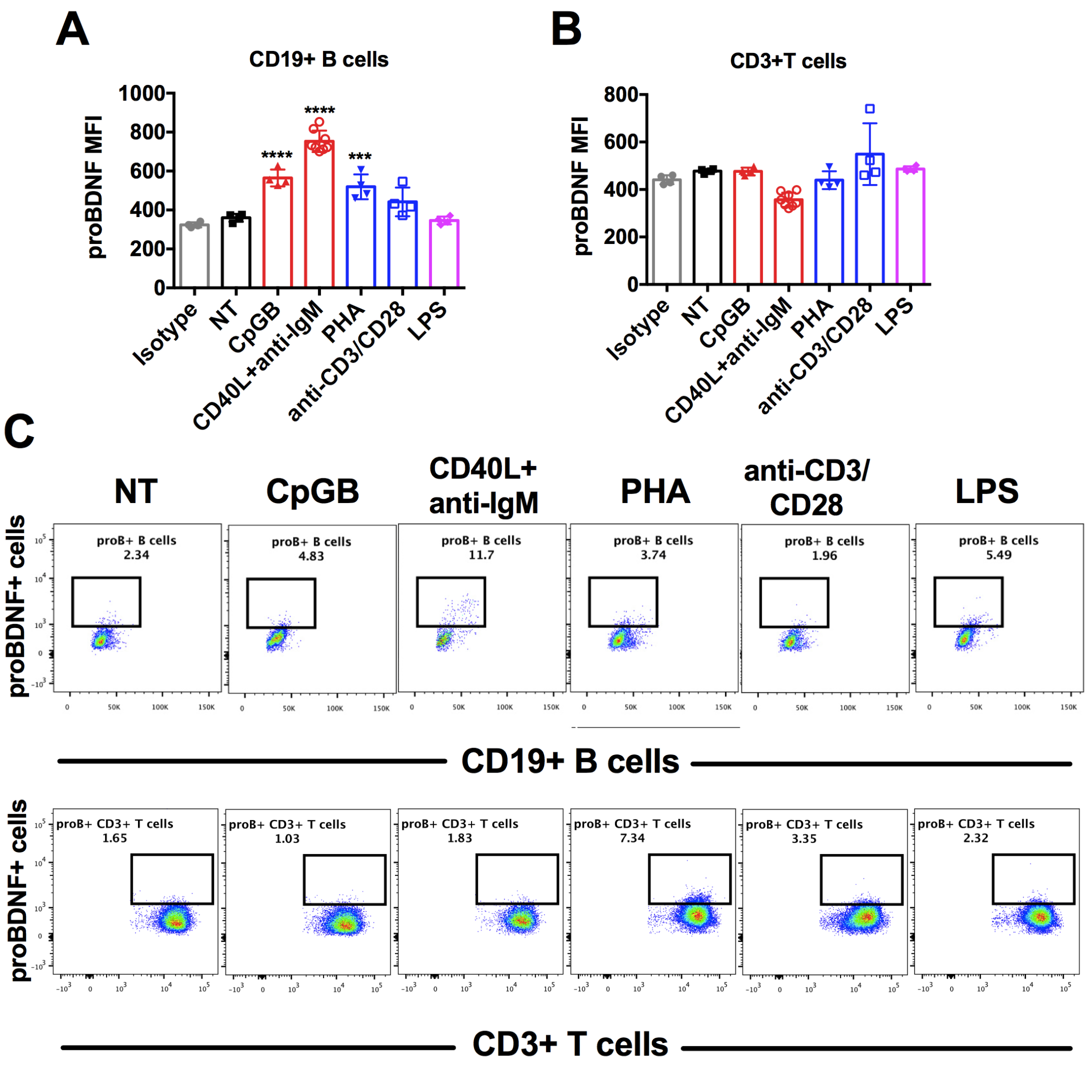

Supplemental Figure 4. ProBDNF expression was elevated in activated B cells in PBMCs derived from healthy donors. PBMCs isolated from healthy donors (HD) were activated for 3 days followed by staining to detect cell surface CD3 and intracellular proBDNF level by flow cytometry. (**A** and **B**). Statistical analysis of proBDNF intracellular flow cytometry showed proBDNF MFI changes in $CD19^+$ B cells (**A**) and $CD3^+$ T cells (**B**) in activated PBMCs. One-way ANOVA followed by Bonferroni's multiple comparison post hoc test, **<0.01 compared to NT, n = 3 in each group, repeated 3 times. (**C**) Representative flow cytometry images showing changes

in percentage of proBDNF$^+$ cells in CD19$^+$ B cells (upper panel) and CD3$^+$ T cells (lower panel) in PBMCs after stimulation with different stimuli for 3 days. Error bars represent mean ± s.e.m. NT: no treatment.

Supplemental Table. 1 General information of human multiple sclerosis patients and healthy donors' PBMCs used in flow cytometry.

	MS	HD	P Value
Sample number	13	16	
Female: Male*	10:3	10:6	0.70
Age (year)	40.31 ± 3.48	42.5 ± 2.68	0.56
Course duration (year)	2.89 ± 0.69	-	-
Age of first-episode (year)	39.63 ± 2.65	-	-
Number of episodes (median)	3 (1~8)	-	-
EDSS score (median)	3.5 (2~8)	-	-

* Chi-square test result

Supplemental Table. 2 Information of human multiple sclerosis patients' brain and spinal cord specimens used in western blot.

MS Brain

Number	Age&sex	MS&duration	COD	PMI	Brain weight at autopsy	Slice	Location
MS50 13A1	68F	SPMS 36 years	hypoxic brain injury	15 hours	1152g	13	Parietal WM
MS59 9B4	70M	SPMS 34 years	MS	25 hours	1480g	9	Parietal WM
MS63 7 A1	41M	SPMS 14 years	MS	17 hours	1267g	7	Superior Frontal
MS62 5B2	47F	MS likely SPMS 26 years	MS	21 hour	1196g	5B2	Occipital deep WM
MS7 8B1	36F	PPM 13 years	bronchopneumonia	24 hours	1168g	8	Parietal deep WM
MS60 18B2	73M	SPMS 16 years	MS	25 hours	1402g	18	Occipital deep WM

MS Spinal cord

Number	Age&sex	MS&duration	COD	PMI	Block	Location
MS59-12	70M	SPMS 34 years	MS	25 hours	Normal cord	Thoracic and lumbar cord
MS50-9	68F	SPMS 36 years	hypoxic brain injury	15 hours	Normal cord	Thoracic and lumbar cord
MS46-1	51F	SPMS 7 years	Sepsis, MRSA	17 hours	MS lesions+some unaffected	Thoracic cord
MS63-7	41M	SPMS 14 years	MS	17 hours	MS lesion+some unaffected area	Lower thoracic cord
MS4-Th3	48M	SPMS 13 years	coronary artery thrombosis	29 hours	MS lesions	Thoracic cord
MS41-13	57F	SPMS 18 years	cardiorespiratory arrest	27 hours	MS lesions	Thoracic cord
MS41-9	57F	SPMS 18 years	cardiorespiratory arrest	27 hours	Near normal	Thoracic cord
MS50-13	68F	SPMS 36 years	hypoxic brain injury	15 hours	MS lesions	Thoracic and lumbar cord
MS59-4	70M	SPMS 34 years	MS	25 hours	MS lesions	Thoracic and lumbar cord
MS4-1	48M	SPMS 13 years	coronary artery thrombosis	29 hours	MS lesions	Thoracic cord
MS62-11	47F	MS likely SPMS 26 years	MS	21 hours	MS lesions+some unaffected	Thoracic cord
MS 16-7	59F	SPMS 28 years	cardiorespiratory arrest	18 hours	MS lesions-Wallerian degeneration	Thoracic cord
MS42-8	64M	SPMS 21 years	acute myocardial infarction	15 hours	Many lesions+normal on same side	Thoracic cord

MS: multiple sclerosis; COD: Cause of death; PMI: postmortem interval, SPMS: secondary-

progressive MS; PPMS: primary progressive MS; WM: white matter.

Supplemental Table. 3 Table. Antibodies and stimulants used in this study

Antigen	Conjugation	Isotype	Catalog	Manufacturer	Use
ProBDNF	-	Rabbit IgG	ANT006	Alomone labs ANT-006	IHC, IF, WB， IP
Sortilin	-	Rabbit IgG	ANT009	Alomone labs ANT-009	IFC, WB
P75 NGF Receptor	-	Rabbit IgG	ab52987	Abcam	WB
GAPDH	-	Rabbit IgG	ab181602	Abcam	WB
MBP	-	Mouse IgG	ab62631	Abcam	IF
ProBDNF	-	Sheep IgG	-	provided by Prof. Zhou	FC
CD3	-	Rat IgG2b	14-0032-81	eBioscience	IF
CD4	-	Rat IgG2b	16-0041-81	eBioscience	IF
CD8	-	Mouse IgG2a	sc-7970	Santa Cruz	IF
CD45R/B220	-	Rat IgG	ab64100	Abcam	IF
P65	-	Rabbit IgG	10745-1-AP	Proteintech	WB
RhoA	-	Rabbit IgG	10749-1-AP	Proteintech	WB
JNK	-	Rabbit IgG	51151-1-AP	Proteintech	WB
P-JNK	-	Rabbit IgG	ab124956	Abcam	WB
human IgG4-Fc Protein	-	-	13505-HNAH	Sino Biological	isotype control
DAPI	-	-	SC-3598	Santa Cruz Biotechnology	IF
rProtein A/G beads	-	-	88802	Invitrogen	IP
**Mouse**	-				
CD3	BV421	hamster anti mouse CD3e	562600	BD Pharmingen	FC
CD4	APC/Cy7	Rat IgG2a	100414	Biolegend	FC
CD8	PE-Cy7	Rat IgG2a	100722	Biolegend	FC
CD19	APC	Rat IgG2a	115512	Biolegend	FC
CD271	PE	-	130-110-113	Miltenyi Biotec	FC

TruStain FcX (anti-mouse CD16/32)	-	-	101309	Biolegend	FC
**Human**					
CD3	PE-Cy7	Mouse BALB/c IgG1	563423	BD Pharmingen	FC
CD4	PerCP-Cy5.5	Mouse IgG2b	317428	Biolegend	FC
CD8	BV510	Mouse BALB/c IgG1	563919	BD Pharmingen	FC
CD19	APC	Mouse BALB/c IgG1	302212	BD Pharmingen	FC
CD271	PE	-	130-110-113	Miltenyi Biotec	FC
Sheep IgG	-	-	ab37385	Abcam	FC
Rabbit IgG	-	-	ab172730	Abcam	FC
Human TruStain FcX	-	-	422302	Biolegend	FC
CpG-B	-	-	ODN2006	Invivogen	cell culture
CD40L	-	-	LAX-522-015-C010	Enzolife science	cell culture
anti-IgM	-	-	A3437	Sigma	cell culture
PHA	-	-	L1668	Sigma	cell culture
anti-CD3/CD28	-	-	11161D	Thermo Fisher Scientific	cell culture
LPS	-	-	L2630	Sigma	cell culture
**Secondary antibody/reagent**		**Conjugation**	**Manufacturer**		**Use**
Goat anti-Rabbit IgG		HRP	Abcam ab205718		WB
Donkey Anti-Mouse IgG		AF594	Abcam ab150108		IF
Donkey Anti-Sheep IgG		FITC	Abcam ab6896		IF
Donkey Anti-Rabbit IgG		FITC	Abcam ab6798		IF

Supplemental Table. 4 The primer sequences used for qPCR in this study

Target gene	Primer sequence
**Mouse**	

IL-1β	S	5'– GAAATGCCACCTTTTGACAGTG –3'
	AS	5'– TGGATGCTCTCATCAGGACAG –3'
IL-6	S	5'– CTCTGGCTTTGTCTTTCTTGTTATCTTT –3'
	AS	5'– AGTTGTGCAATGGCAATTCTGA –3'
IL-17	S	5'– CCTCAGACTACCTCAACCGTTCC –3'
	AS	5'– GTGGTGGTCCAGCTTTCCCT –3'
TNF-α	S	5'– AGGCGGTGCCTATGTCTCAG –3'
	AS	5'– GCTCCTCCACTTGGTGGTTT –3'
IFN-γ	S	5'– CCTCTCATGCACCACCATCA –3'
	AS	5'– GCATTGCACCTCAGGGAAGA –3'
GAPDH	S	5'– CTGCCCAGAACATCATCCCT –3'
	AS	5'– TGGTCCTCAGTGTAGCCCAAG –3'
**Human**		
IL-1β	S	5'– CCTGAGCTCGCCAGTGAAAT–3'
	AS	5'– TCGTGCACATAAGCCTCGTT –3'
IL-6	S	5'– ATGTAGCCGCCCCACACAGA –3'
	AS	5'– CATCCATCTTTTTCAGCCAT –3'
GM-CSF	S	5'– GGATGTGGCTGCAGAGCCTGC–3'
	AS	5'– CTGGATGGCATTCACAT–3'
TNF-α	S	5'– GCTGCACTTTGGAGTGATCG –3'
	AS	5'– ATGAGGTACAGGCCCTCTGA –3'
β-Actin	S	5'– CATGTACGTTGCTATCCAGGC –3'
	AS	5'– CTCCTTAATGTCACGCACGAT –3'
